# Quantitative Lesion-to-Fat Elasticity Ratio Measured by Shear-Wave Elastography for Breast Mass: Which Area Should Be Selected as the Fat Reference?

**DOI:** 10.1371/journal.pone.0138074

**Published:** 2015-09-14

**Authors:** Ji Hyun Youk, Eun Ju Son, Hye Mi Gweon, Kyung Hwa Han, Jeong-Ah Kim

**Affiliations:** 1 Department of Radiology, Gangnam Severance Hospital, Yonsei University College of Medicine, Seoul, Republic of Korea; 2 Biostatistics Collaboration Unit, Gangnam Medical Research Center, Yonsei University College of Medicine, Seoul, Republic of Korea; Fondazione IRCCS Istituto Nazionale dei Tumori, ITALY

## Abstract

**Objectives:**

To investigate whether the diagnostic performance of lesion-to-fat elasticity ratio (E_ratio_) was affected by the location of the reference fat.

**Methods:**

For 257 breast masses in 250 women who underwent shear-wave elastography before biopsy or surgery, multiple E_ratio_s were measured with a fixed region-of-interest (ROI) in the mass along with multiple ROIs over the surrounding fat in different locations. Logistic regression analysis was used to determine that E_ratio_ was independently associated with malignancy adjusted for the location of fat ROI (depth, laterality, and distance from lesion or skin). Mean (E_mean_) and maximum (E_max_) elasticity values of fat were divided into four groups according to their interquartile ranges. Diagnostic performance of each group was evaluated using the area under the ROC curve (AUC). False diagnoses of E_ratio_ were reviewed for ROIs on areas showing artifactual high or low stiffness and analyzed by logistic regression analysis to determine variables (associated palpable abnormality, lesion size, the vertical distance from fat ROI to skin, and elasticity values of lesion or fat) independently associated with false results.

**Results:**

E_ratio_ was independently associated with malignancy adjusted for the location of fat ROI (P<0.0001). Among four groups of fat elasticity values, the AUC showed no significant difference (<25th percentile, 25th percentile~median, median~75th percentile, and ≥75th percentile; 0.973, 0.982, 0.967, and 0.954 for E_mean_; 0.977, 0.967, 0.966, and 0.957 for E_max_). Fat elasticity values were independently associated with false results of E_ratio_ with the cut-off of 3.18 from ROC curve (P<0.0001). ROIs were set on fat showing artifactual high stiffness in 90% of 10 false negatives and on lesion showing vertical striped artifact or fat showing artifactual low stiffness in 77.5% of 71 false positives.

**Conclusion:**

E_ratio_ shows good diagnostic performance regardless of the location of reference fat, except when it is placed in areas of artifacts.

## Introduction

Breast elastography as a method of imaging tissue stiffness has been used to improve diagnostic confidence and increase the specificity of ultrasound interpretation. The recently developed shear-wave elastography (SWE) uses the acoustic radiation force induced by the ultrasound push pulse generated by the ultrasound transducer [1]. This force induces mechanical waves, including shear waves, which propagate transversely in the tissue. The SWE allows measurement of the propagation speed of shear waves within the tissue to locally quantify its stiffness in kilopascals (kPa). Within a given region of interest (ROI), a variety of stiffness parameters can be measured, including the mean stiffness (E_mean_), maximum stiffness (E_max_), and standard deviation [2].

In addition, SWE can provide the elasticity ratio (E_ratio_) of the breast lesion to the reference fat tissue, similar to the strain ratio obtained from strain elastography technique. In previous studies, the diagnostic performance of E_ratio_ has been as good as that of E_mean_ or E_max_ [3–5], or even the highest among SWE parameters [6–8]. In measuring E_ratio_, however, there has been no precise information about the location of ROI for the reference fat, although elasticity values of breast mass such as E_mean_ and E_max_ are supposed to be measured over the stiffest part of the lesion including the immediate adjacent stiff tissue or halo. Considering the ultrasonographic breast anatomy that the layers of subcutaneous and retromammary fat are over and beneath the fibroglandular tissue, respectively, and intervened with the fibroglandular tissue, the ROI for the reference can be set on various locations in measuring E_ratio_. For strain elastography, previous studies reported that changing the position of the reference area influenced the strain ratio measurements [9,10]. However, there has been no study of the position of the reference when measuring E_ratio_ for breast mass at SWE.

Therefore, this study was performed to investigate whether the diagnostic performance of E_ratio_ was affected by the location of ROI in the reference fat.

## Materials and Methods

The present retrospective study was conducted with institutional review board approval of Gangnam Severance Hospital and a waiver of patient informed consent from the participants. All patient records/information was anonymized and de-identified prior to analysis.

Between February 2013 and August 2013, 363 consecutive women who had been scheduled to undergo ultrasound-guided core needle biopsy or surgical excision for breast masses were examined by SWE. Among these patients, 257 breast masses in 250 women aged 22–90 years (mean, 47.1 ± 11.1 years) were enrolled in the current study. The remaining 113 women were excluded from the present study because multiple quantitative E_ratio_s measured for the breast mass were not available.

### Ultrasound Examinations

**Fig 1:**
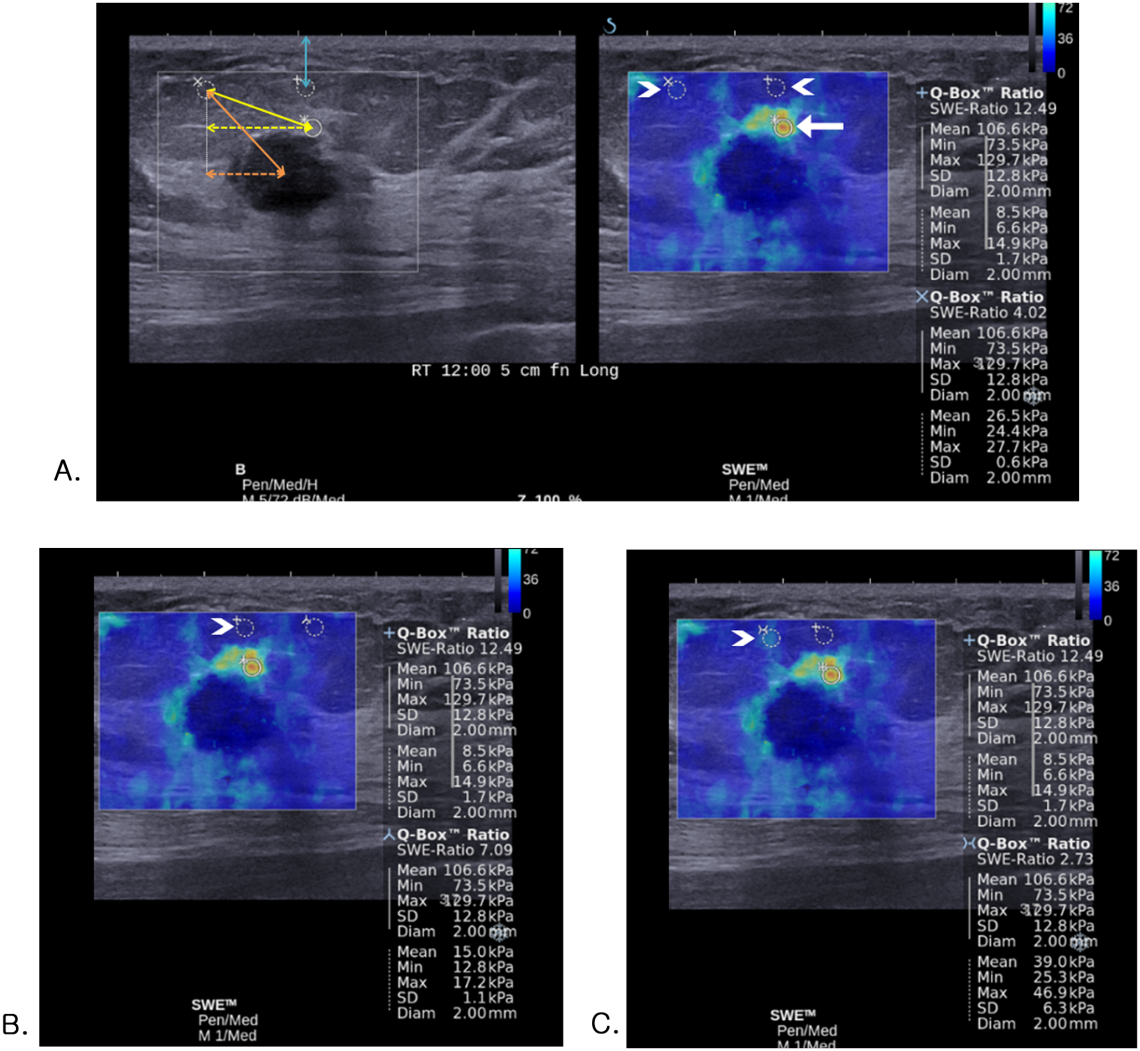
Images of invasive ductal carcinoma in a 54-year-old woman. (A-C) At a single SWE image obtained from the mass, four E_ratio_s are measured with a fixed ROI for the mass (white arrow) along with four ROIs for the surrounding fat that are set randomly in different four locations (white arrowheads). (A, left) The actual (orange double-headed solid arrow) or vertical (orange double-headed dotted arrow) distance from the center of lesion to the fat ROI, the actual (yellow double-headed solid arrow) or vertical (yellow double-headed dotted arrow) distance from the lesion ROI to the fat ROI, and the vertical distance from the fat ROI to skin (blue double-headed solid arrow) was measured on gray-scale image. (C) For the ROI that is set on the fat tissue showing artifactual vertical light blue color stiffness at SWE (E_mean_, 39.0 kPa; E_max_, 46.9 kPa) (white arrowhead), E_ratio_ was 2.73 which was false negative result according to the cutoff value of 3.18.

Breast ultrasound examinations were performed using the Aixplorer ultrasound system (SuperSonic Imagine, Aix-en-Provence, France), which was equipped with a 4–15-MHz linear-array transducer, by one of four radiologists with 5–10 years of experience in breast ultrasound. The investigators knew the clinical examination and mammography results at the time of the ultrasound examination. After obtaining gray-scale ultrasound, SWE images were obtained for the breast masses that were scheduled to be biopsied or excised surgically. The built-in region-of-interest (ROI) (Q-box; SuperSonic Imagine) of the system was set to include the lesion and surrounding normal tissue, which demonstrated that a semitransparent color map of tissue stiffness overlaid the gray-scale image with a range from dark blue, indicating the lowest stiffness up to red, indicating the highest stiffness (0–180 kPa). Areas of black on the SWE images represented tissue in which no shear wave was detected. Fixed 2 x 2-mm ROIs were placed by an investigator over the stiffest part of the lesion, including immediate adjacent stiff tissue or halo. A second ROI of the same size was placed in the breast fatty tissue. This allowed calculation of the ratio between the mean elasticity values in the lesion and in the fat, E_ratio_, by the ultrasound system. At a single SWE image obtained from each mass, at least two quantitative E_ratio_s were measured with a fixed ROI for the mass along with ROIs for the surrounding fat that were set randomly in different locations during the SWE examination (Fig 1). The system calculated automatically E_max_ and E_mean_ in kPa as well as E_ratio_ for the mass.

### Image Evaluation and Data Analysis

For each SWE image, the location of each ROI for the fat was evaluated on a Picture Archiving and Communication System as follows: depth (superficial, deep, or equal to the center of the lesion ROI), laterality (right, left, or center to the lesion ROI), the actual or vertical distance from the center of lesion to the fat ROI (mm), the actual or vertical distance from the lesion ROI to the fat ROI (mm), and the vertical distance from the fat ROI to skin (mm) (Fig 1).

The patients’ medical records were reviewed and data on the lesion size and the associated palpable abnormality were compiled. With random effect model, the mean of E_ratio_ was compared between benign and malignant masses. Logistic regression analysis with random effect was used to determine that E_ratio_ was independently associated with breast cancers adjusted for the location of ROI for the fat (depth, laterality, the actual or vertical distance from lesion, the actual or vertical distance from the ROI of lesion, and the vertical distance from skin). Quantitative E_mean_ and E_max_ of the reference fat were divided into four groups according to their interquartile range (< 25th percentile, 25th percentile ~ median, median ~ 75th percentile, and ≥ 75th percentile). Diagnostic performance of E_ratio_ in each group was evaluated and compared using the area under the receiver operating characteristic (ROC) curve (AUC) by the DeLong method [11] to determine whether the diagnostic performance of E_ratio_ was different according to E_mean_ and E_max_ of the reference fat. False negative or false positive results according to the optimal cut-off value for E_ratio_ calculated from ROC curve were analyzed by logistic regression analysis with random effect to determine variables (the associated palpable abnormality, lesion size, the vertical distance from fat ROI to skin, and elasticity values of lesion or fat) independently associated with false results of E_ratio_. SWE images of false results were reviewed by four radiologists in consensus to determine whether ROIs were set on lesion or fat tissue showing artifactual high or low stiffness.

Statistical analysis was performed using statistical software programs (SAS, version 9.2, SAS Institute Inc., Cary, NC, USA). Differences were considered to be statistically significant for a P value less than 0.05.

## Results

Of the 257 masses, 122 (47.5%) were malignant and 135 (52.5%) were benign. Lesion size defined as the maximal diameter at ultrasound ranged from 3 to 38 mm (mean, 14.5 ± 8.2 mm). The mean lesion size of benign masses was 11.9 mm (range, 3–35 mm) and that of malignant masses was 17.6 mm (range, 4–38 mm). 71 masses (27.6%) were palpable. On per-case basis, the number of E_ratio_ measured for each breast mass ranged from two to seven (mean, 4.3) and there was 1,109 E_ratio_ measurements after including all the measurements for each lesion. The mean of E_ratio_ was significantly different between benign (2.33 ± 1.84) and malignant masses (8.39 ± 5.51) (P < 0.0001). For the diagnosis of malignant breast mass, the odds ratio of E_ratio_ was 1.988 (95% confidence interval (CI), 1.715 to 2.305). In the ROC curve analysis, the AUC of E_ratio_ was 0.926 (95% CI, 0.911 to 0.941) with the optimal cut-off value of 3.18 (sensitivity, 91.5%; specificity, 79.9%). The optimal cut-off value of lesion E_mean_ and E_max_ was 59.8 kPa (95% CI, 55.7 to 80.6) and 78.2 kPa (95% CI, 72.9 to 108.6), respectively.

For the location of fat ROI in 1,109 cases of E_ratio_, the depth of fat ROI was equal in 185 cases (16.7%), superficial in 893 cases (80.5%), and deep in 31 cases (2.8%) to lesion ROI. The laterality of fat ROI was center in 123 cases (11.1%), right in 426 cases (38.4%), and left in 560 cases (50.5%) to lesion ROI. The means were 11.9 mm of the actual distance from lesion center (range, 1.2–25.8 mm), 9.0 mm of the vertical distance from lesion center (range, 0.3–24.3 mm), 10.8 mm of the actual distance from lesion ROI (range, 2.6–23.9 mm), 8.9 mm of the vertical distance from lesion ROI (range, 0.2–22.3 mm), and 5.6 mm of the vertical distance from skin (range, 1.1–23.2 mm). At logistic regression analysis, higher E_ratio_ was independently associated with breast cancer adjusted for the location of ROI in fat (depth, laterality, the actual or vertical distance from the lesion, the actual or vertical distance from the ROI of lesion, and the vertical distance from skin) (P < 0.0001). The AUC of E_ratio_ was not changed according to the location of ROI in the fat (Table 1).

**Table 1:** Diagnostic performance of E_ratio_ for characterization of breast masses, adjusted for location of fat ROI.

Model	Variable	Odds ratio (95% CI)	P value	AUC
**E**_**ratio**_	**E**_**ratio**_	1.988 (1.715, 2.305)	< 0.0001	0.926
**+ Depth** *	**E**_**ratio**_	1.942 (1.671, 2.257)	< 0.0001	0.927
	**Equal vs Superficial**	0.448 (0.167, 1.197)	0.109	
	**Equal vs Deep**	0.921 (0.074, 11.504)	0.949	
**+ Laterality** *	**E**_**ratio**_	2.010 (1.729, 2.337)	< 0.0001	0.926
	**Center vs Right**	0.692 (0.248, 1.931)	0.482	
	**Center vs Left**	0.918 (0.338, 2.492)	0.866	
**+ Actual distance from lesion center**	**E**_**ratio**_	1.969 (1.695, 2.288)	< 0.0001	0.926
	**Distance (mm)**	1.031 (0.947, 1.123)	0.476	
**+ Vertical distance from lesion center**	**E**_**ratio**_	1.989 (1.709, 2.314)	< 0.0001	0.926
	**Distance (mm)**	0.999 (0.932, 1.072)	0.988	
**+ Actual distance from lesion ROI**	**E**_**ratio**_	1.997 (1.720, 2.318)	< 0.0001	0.926
	**Distance (mm)**	0.979 (0.906, 1.057)	0.584	
**+ Vertical distance from lesion ROI**	**E**_**ratio**_	1.985 (1.708, 2.307)	< 0.0001	0.926
	**Distance (mm)**	1.004 (0.938, 1.074)	0.913	
**+ Vertical distance from skin**	**E**_**ratio**_	1.994 (1.718, 2.314)	< 0.0001	0.927
	**Distance (mm)**	0.957 (0.823, 1.113)	0.569	

AUC = the area under the receiver operating characteristic curve, CI = Confidence interval, E_ratio_ = elasticity ratio, the ratio between the mean elasticity values in the lesion and in the fatty tissue, ROI = Region of interest

* Relative location of the ROI for fat to the ROI for lesion

Regarding the elasticity values of the reference fat, the 25th percentile, median, and 75th percentile of elasticity values of the reference fat were 11.5 kPa, 16.9 kPa, and 25.3 kPa for E_mean_ (range, 1.7–59.9 kPa) and 16.3 kPa, 24.7 kPa, and 35.7 kPa for E_max_ (range, 2.6–95.2 kPa), respectively. There was no significant difference in the AUC of E_ratio_ according to the interquartile range of the elasticity values of the reference fat (Table 2).

**Table 2:** Diagnostic performance of E_ratio_ according to the interquartile range of elasticity value of the fat.

Fat elasticity value	IQR	AUC of E_ratio_ (95% CI)	Comparison of AUC of E_ratio_	P value
**E**_**mean**_	**< 25** ^**th**^ **percentile**	0.973 (0.954, 0.991)	**< 25** ^**th**^ **percentile vs 25** ^**th**^ **percentile ~ median**	0.415
	**25** ^**th**^ **percentile ~ median**	0.982 (0.969, 0.996)	**< 25** ^**th**^ **percentile vs Median ~ 75** ^**th**^ **percentile**	0.694
	**Median ~ 75** ^**th**^ **percentile**	0.966 (0.942, 0.991)	**< 25** ^**th**^ **percentile vs ≥ 75** ^**th**^ **percentile**	0.283
	**≥ 75** ^**th**^ **percentile**	0.954 (0.926, 0.982)	**25** ^**th**^ **percentile ~ median vs Median ~ 75** ^**th**^ **percentile**	0.267
			**25** ^**th**^ **percentile ~ median vs ≥ 75** ^**th**^ **percentile**	0.077
			**Median ~ 75** ^**th**^ **percentile vs ≥ 75** ^**th**^ **percentile**	0.436
**E**_**max**_	**< 25** ^**th**^ **percentile**	0.977 (0.961, 0.992)	**< 25** ^**th**^ **percentile vs 25** ^**th**^ **percentile ~ median**	0.462
	**25** ^**th**^ **percentile ~ median**	0.967 (0.947, 0.987)	**< 25** ^**th**^ **percentile vs Median ~ 75** ^**th**^ **percentile**	0.449
	**Median ~ 75** ^**th**^ **percentile**	0.966 (0.942, 0.989)	**< 25** ^**th**^ **percentile vs ≥ 75** ^**th**^ **percentile**	0.196
	**≥ 75** ^**th**^ **percentile**	0.957 (0.930, 0.983)	**25** ^**th**^ **percentile ~ median vs Median ~ 75** ^**th**^ **percentile**	0.925
			**25** ^**th**^ **percentile ~ median vs ≥ 75** ^**th**^ **percentile**	0.531
			**Median ~ 75** ^**th**^ **percentile vs ≥ 75** ^**th**^ **percentile**	0.591

IQR = Interquartile range, AUC = the area under the receiver operating characteristic curve, CI = Confidence interval, E_mean_ = mean elasticity value of the reference fat, E_max_ = maximum elasticity value of the reference fat, E_ratio_ = elasticity ratio, the ratio between the mean elasticity values in the lesion and in the fatty tissue

**Fig 2:**
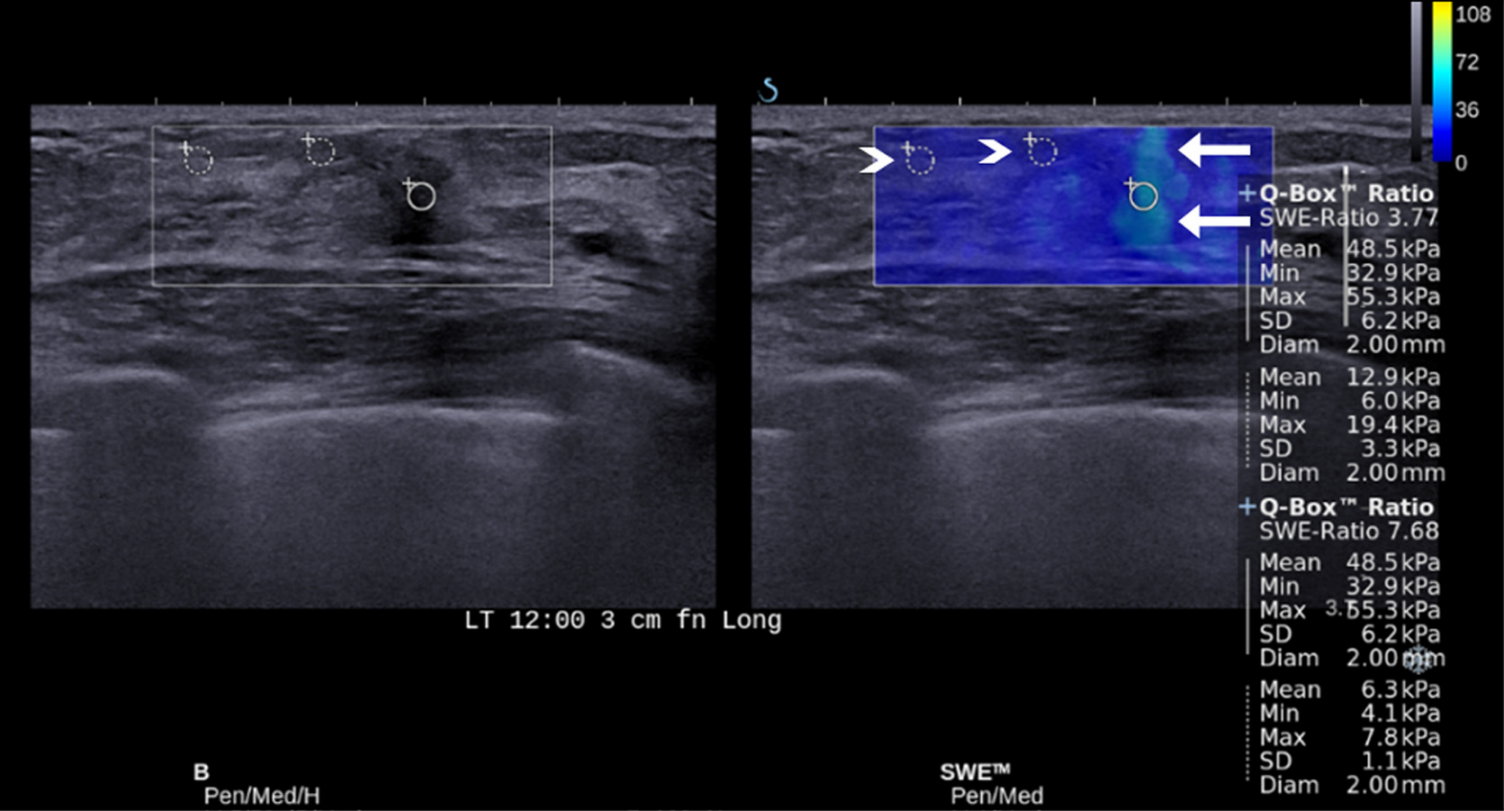
Images of fibrocystic change in a 43-year-old woman. For the ROI that is set on the lesion showing the vertical stripe pattern of artifacts (arrows) at SWE (E_mean_, 48.5 kPa; E_max_, 55.3 kPa), E_ratio_s were 3.77 and 7.68 measured with two different fat ROIs (arrowheads) which were false positive results according to the cutoff value of 3.18.

**Fig 3:**
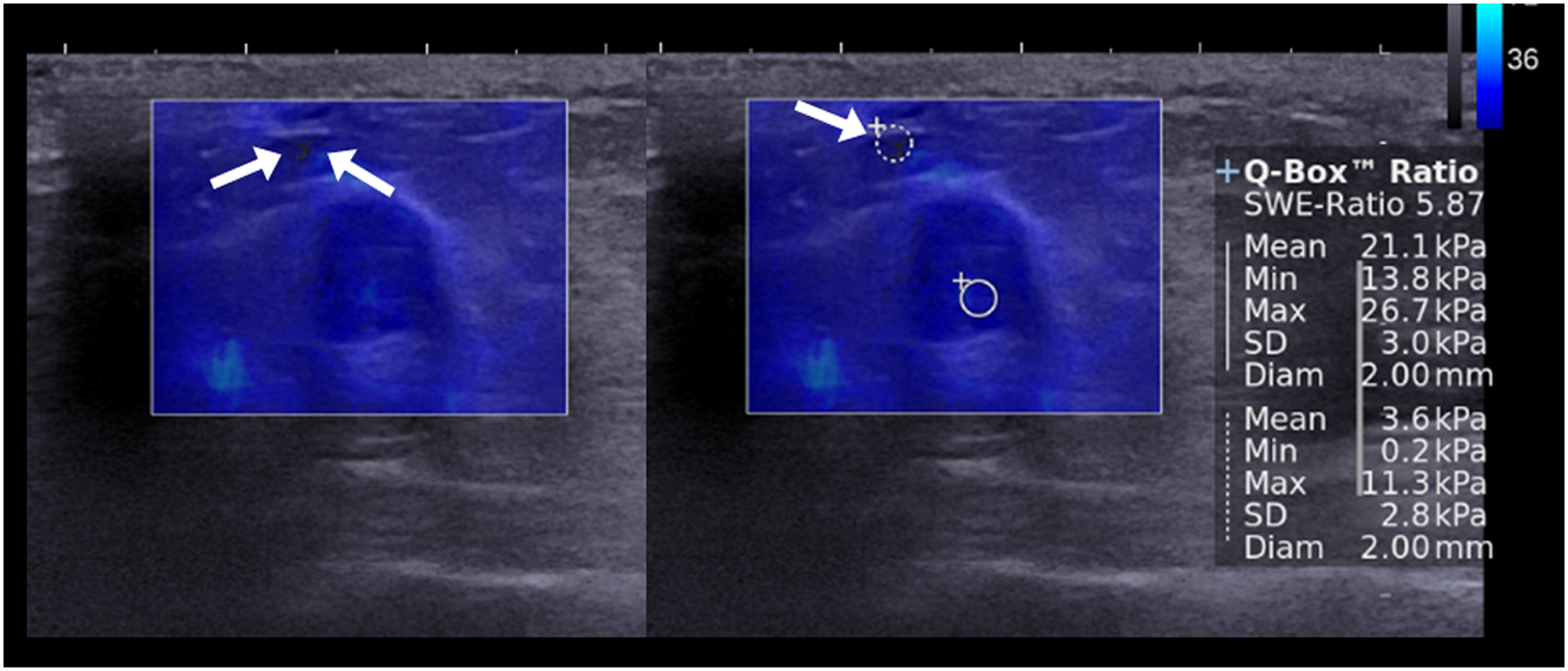
Images of fibroadenoma in a 42-year-old woman. For the ROI that is set on the fat tissue showing black color at SWE (E_mean_, 3.6 kPa; E_max_, 11.3 kPa) (arrows), E_ratio_ was 5.87 which was false positive result according to the cutoff value of 3.18.

Among 1,109 cases of E_ratio_, 110 (9.9%) were false positive and 48 (4.3%) were false negative according to the cut-off value of 3.18 from ROC curve. On per-lesion basis, E_ratio_s obtained from a single mass were all true in 160 masses, all false negative in one mass, and all false positive in one mass. In the remaining 95 masses, both true and false results (false positive in 66 and false negative in 29) were obtained from the same mass with the different fat ROI. Table 3 provides the variables independently associated with false positive or false negative results of E_ratio_. For false positive results, the odds significantly increased with associated palpable abnormality (P = 0.0004) and higher elasticity values of lesion and significantly decreased with higher elasticity values of fat (P < 0.0001). For false negative results, the odds significantly increased with higher elasticity values of fat (P < 0.0001) and significantly decreased with associated palpable abnormality (P = 0.027), and higher elasticity values of lesion (P < 0.0001). For E_mean_ and E_max_ of fat, 10 false negative cases and 71 false positive cases were found after adjusting associated palpable abnormality, with the cut-off values of 59.8 kPa in lesion E_mean_ and 78.2 kPa in lesion E_max_. After reviewing SWE images of those 81 false cases for artifacts, ROIs were set on fat tissue showing artifactual high stiffness in nine (90%) of 10 false negative cases (Fig 1) and on lesion showing vertical striped artifact (n = 48; 67.6%) (Fig 2) or fat tissue showing artifactual low stiffness (n = 7; 9.9%) (Fig 3) in 55 (77.5%) of 71 false positive cases.

**Table 3:** Logistic regression analysis of variables independently associated with false positive or negative results of E_ratio_.

E_ratio_	Variable	Odds ratio (95% CI)	P value
**False positive**	**Palpability**	3.167 (1.679, 5.975)	0.0004
	**Lesion size (mm)**	1.025 (0.993, 1.059)	0.127
	**Distance from skin (mm)**	1.028 (0.918, 1.150)	0.633
	**Lesion E**_**mean**_ **(kPa)**	1.059 (1.044, 1.075)	<0.0001
	**Lesion E**_**max**_ **(kPa)**	1.045 (1.033, 1.057)	<0.0001
	**Fat E**_**mean**_ **(kPa)**	0.849 (0.813, 0.886)	<0.0001
	**Fat E**_**max**_ **(kPa)**	0.857 (0.827, 0.889)	<0.0001
**False negative**	**Palpability**	0.360 (0.145, 0.889)	0.027
	**Lesion size (mm)**	0.946 (0.904, 1.091)	0.059
	**Distance from skin (mm)**	0.876 (0.734, 1.045)	0.141
	**Lesion E**_**mean**_ **(kPa)**	0.967 (0.958, 0.975)	<0.0001
	**Lesion E**_**max**_ **(kPa)**	0.972 (0.965, 0.979)	<0.0001
	**Fat E**_**mean**_ **(kPa)**	1.134 (1.097, 1.173)	<0.0001
	**Fat E**_**max**_ **(kPa)**	1.091 (1.063, 1.120)	<0.0001

E_ratio_ = elasticity ratio, the ratio between the mean elasticity values in the lesion and in the fatty tissue, CI = Confidence interval, E_mean_ = mean elasticity value, E_max_ = maximum elasticity value, Distance from skin = vertical distance from skin to ROI for the fat

## Discussion

In the present study, we analyzed the diagnostic performance of E_ratio_ at SWE according to the different location of ROI in fat (depth, laterality, the distance from the lesion, and the distance from skin). We discovered that the diagnostic performance of E_ratio_ was not influenced by the measurement site of fat elasticity and the odds of breast cancer significantly increased with higher E_ratio_, regardless of the location of ROI in fat (Table 1). Although there is no report of E_ratio_ at SWE according the location of the reference fat, a few studies have reported the relationship between the lesion depth and the lesion stiffness. In a phantom study of tissue quantification using acoustic radiation force impulse technology from another ultrasound system of SWE, shear wave velocity measured at varying depths of the target regions showed the increasing dispersion of the values for the deep target regions [12]. Similarly, a study of SWE with 939 breast masses using the prototype ultrasound system of SWE reported that there was a 4.5-kPa decrease in lesion stiffness for each 5-mm increase in lesion depth (P = 0.01), on average, across all masses [3]. At SWE, the ultrasound push pulse generated by the ultrasound transducer is attenuated as it traverses tissue, which can result in a reduction of the amplitude of shear waves generated in the deeper lesion [13]. In the present study, the diagnostic performance of E_ratio_ was evaluate according to the location of the fat ROI, unlike those previous studies that evaluated the lesion elasticity values according to the lesion depth from the skin, which could explain the discrepancy in results. Interestingly, a previous study reported that the discriminating ability of E_ratio_ in malignancy was not influenced by the lesion depth from skin [8]. Further evaluation may be necessary to find out how the discriminating ability of E_ratio_ in malignancy was not influenced by the depth from the lesion or the reference fat to skin.

For E_ratio_ at SWE, the elasticity value of the reference fat can be as important as the lesion stiffness is, rather than the location of the fat ROI itself, because the fat stiffness is the denominator in calculating E_ratio_ of the lesion arithmetically. Even small difference in fat stiffness could be expected to make a difference in E_ratio_ significant. In the present study, E_mean_ and E_max_ of the reference fat showed wide variability, ranging from 1.7 to 59.9 kPa and from 2.6 to 95.2 kPa, respectively, which was attributable to fat ROIs that were set randomly. However, the 25th percentile, median, and 75th percentile of elasticity values of the reference fat except for the 75th percentile of E_max_ (35.7 kPa) were around the range of reported normal fat values (18 to 24 kPa) in the literature [14]. Moreover, all groups of E_mean_ and E_max_ of the reference fat according to their interquartile range showed good diagnostic performance of E_ratio_ with AUC ranging from 0.954 to 0.982. Although the group of 75th percentile or more showed the lowest AUC, the diagnostic performance of E_ratio_ was not significantly different among the groups (Table 2).

The results of the current study showed a good diagnostic performance of E_ratio_, regardless of the location of fat ROI and the elasticity values measured in fat. However, false negative or positive results of E_ratio_ still remain as a pitfall in the diagnosis of breast cancer. We found 95 masses (37%, of 257) showing both true and false results (false positive in 66 and false negative in 29) according to the different fat ROI, which were reviewed and analyzed to determine associated features. During the time of obtaining SWE images, artifactual high or low strain can be seen in the surrounding fat tissue by factors affecting data quality and producing speed errors or loss of signal [15]. For example, continuous high strain without connection to the strain from the mass can be shown at the surrounding tissue, or discontinuous high strain can be located at the superficial or deep portion of the ROI box. In a study, SWE images showing such artifactual stiffness were approximately 15.3% [16]. In contrast, some areas with low ultrasonic echo signal in the ROI box can be coded as very dark blue or black color with or without low echo signal at corresponding grey-scale image (Fig 3). As SWE feature of breast lesion, area of black color inside the lesion without elasticity information is well known as black hole phenomenon. It appears as the shear wave cannot propagate into the hard lesion such as malignancy or into liquid areas such as cyst [2,17–19]. For breast fat tissue, such artifactual low stiffness has not been discussed in literatures. Yet, we found seven cases of ROIs set on fat tissue showing artifactual low stiffness (9.9%) among 71 false positive cases. Although we have not found a clear explanation for this finding, factors of shear-wave scattering or mechanical disturbance at tissue boundaries are presumed to reduce or prevent shear-wave penetration in fatty tissue [15]. The artifactual high or low strain in the fat tissue can give us inaccurate elasticity information in E_ratio_. In our study, ROIs were set on fat tissue showing the artifactual high stiffness in 90% of false negative cases and on fat tissue showing the artifactual low stiffness in 9.9% of false positive cases. In multivariate analysis, the odds for false negative results increased with higher elasticity values of fat (P < 0.0001) and the odds for false positive results decreased with higher elasticity values of fat (P < 0.0001). In addition, for lesion elasticity value in E_ratio_, 67.6% of false positive cases were observed in lesions showing vertical striped artifact where lesion ROI was set. Multivariate analysis showed that the odds for false positive results increased with higher elasticity values of lesion (P < 0.0001) (Fig 3). In previous studies, the vertical striped artifact was reported from 13.7% to 30.3% and could be one of causes for erroneous interpretation [4,8,17,20,21]. This artifact is unintentional artifactual vertical bands of stiffness that is observed at the margin or in the interior of the lesion, extends beyond the lesion, and continues vertically in cords on the cutaneous side or the thoracic wall side [17]. These distinctive elastographic features could help identify and avert areas of artifactual high stiffness from setting ROI. It is recommended that compression or movement of the probe be minimized for SWE using generous amounts of contact jelly, the range of Q-box be adjusted to exclude the skin and chest wall layers, and having the patient hold her breath be effective in some cases to reduce artifacts [2]. To avoid false positive or negative results in E_ratio_, therefore, images with good quality should be obtained and ROIs for the lesion and the surrounding fat should be set on the area without artifacts.

The present study has some limitations. Owing to its retrospective nature, there might have been selection bias because patients included in the study were scheduled for biopsy or surgery of known breast lesions, and it was not possible to control the consistent positioning of the fat ROI. For large breast mass, setting multiple ROIs in the surrounding fat tissue might be limited when the mass was big enough to occupy considerable portion of the Q-box for SWE. SWE was performed by one of four radiologists and the interobserver variability could be a limitation. Considering the prior result showing that SWE was highly reproducible for assessing the elastographic features of breast lesions, interobserver variability was expected to have little influence on our result [22].

In conclusion, E_ratio_ shows good diagnostic performance regardless of the location of reference fat, except when it is placed in areas of artifacts. To avoid false diagnosis in E_ratio_, fat ROI should be set on the area without artifactual high or low stiffness.
